# Combined Exposure to Multiple Endocrine Disruptors and Uterine Leiomyomata and Endometriosis in US Women

**DOI:** 10.3389/fendo.2021.726876

**Published:** 2021-08-20

**Authors:** Yuqing Zhang, Yingying Lu, Huiyuan Ma, Qing Xu, Xiaoli Wu

**Affiliations:** ^1^Department of Women Health Care, Women’s Hospital of Nanjing Medical University, Nanjing Maternity and Child Health Care Hospital, Nanjing, China; ^2^Department of Obstetrics and Gynecology, Women’s Hospital of Nanjing Medical University, Nanjing Maternity and Child Health Care Hospital, Nanjing, China

**Keywords:** uterine leiomyomata, endometriosis, mixed exposure, weighted quantile sum (WQS) regression, Bayesian kernel machine regression (BKMR)

## Abstract

**Background:**

Uterine leiomyomata (UL) and endometriosis (EM) are common gynecological diseases damaging the reproductive health of fertile women. Among all the potential factors, environmental endocrine-disrupting chemicals are insufficiently addressed considering the multiple pollutants and mixture exposure.

**Methods:**

Women aged 20 to 54 years old in the National Health and Nutrition Examination Survey (NHANES) 2001-2006, having a complete measurement of ten commonly exposed endocrine-disrupting chemicals (including urinary phthalate metabolites, equol, and whole blood heavy metals) and answered questions about UL and EM were included (N=1204). Multivariable logistic regression model, weighted quantile sum (WQS) regression, and Bayesian kernel machine regression (BKMR) models were implemented to analyze the combined effect of chemicals on the overall association with UL and EM.

**Results:**

In single chemical analysis, equol (OR: 1.90, 95% CI: 1.11, 3.27) and mercury (Hg) (OR: 1.91, 95% CI: 1.14, 3.25) were found positively associated with UL in tertile 3 *vs*. tertile 1. In WQS regression and BKMR models, the significant positive association between WQS index and UL (OR: 2.54, 95% CI: 1.52, 4.29) was identified and the positive relationship between equol and Hg exposure and UL were further verified. Besides, the mixture evaluation models (WQS and BKMR) also found MEHP negatively associated with UL. Although none of the single chemicals in tertile 3 were significantly associated with EM, the WQS index had a marginally positive association with EM (OR: 2.01, 95% CI: 0.98, 4.15), and a significant positive association was identified in subanalysis with participants restricted to premenopausal women (OR: 2.18, 95% CI: 1.03, 4.70). MIBP and MBzP weighted high in model of EM and MEHP weighted the lowest.

**Conclusion:**

Comparing results from these three statistical models, the associations between equol, Hg, and MEHP exposure with UL as well as the associations of MIBP, MBzP, and MEHP exposure with EM warrant further research.

## Introduction

Uterine leiomyomata (UL) and endometriosis (EM) are common gynecological diseases, which affect 20-77% and 5-10% of fertile women, respectively (1, 2). Their symptoms vary from dysmenorrhea, chronic pelvic pain, and irregular uterine bleeding to an increased risk for infertility. Uterine leiomyomata and endometriosis represent a major public health issue (3, 4). Epidemiologic studies indicate many factors associated with uterine leiomyomata and endometriosis, such as age, race, family history, dietary, and lifestyle issues (5, 6). However, the contribution of environmental exposure to these diseases hasn’t been sufficiently addressed. Among the chemicals, the exposure of endocrine-disrupting chemicals (EDCs) raised great concern as their potential disturbance on ovarian steroid hormone. The chemicals disrupting the balance of female hormones could play a role in the development of uterine leiomyomata and endometriosis (7, 8).

Phthalates are commonly used in plastic products, such as food packages, toys, and building materials, to improve their flexibility and elasticity (9). The fertile women are exposed to these chemicals *via* contaminated food and water, inhalation of polluted air, and the usage of cosmetic and personal care products. Some *in vivo* and *in vitro* studies reported the endocrine-disrupting effect of phthalates on the pituitary ovarian axis and their similar function to estrogen (10), which indicates their potential associations with female reproduction health. In addition to man-made chemicals including phthalates, some natural metals including lead (Pb), cadmium (Cd), and mercury (Hg) also have endocrine-disrupting properties, which are considered to have estrogenic activity, and therefore they are also classified as EDCs (11, 12). With their decades-long half-life in the environment, their health effects on female reproduction also need to be addressed. Exogenous estrogen phytoestrogen commonly exists in plants like soy products, nuts, and oilseeds. Daidzein is one kind of phytoestrogen consumed from food made with soybeans and other legumes. As the end product of the biotransformation of daidzein, equol possesses estrogenic activity and was reported to be associated with female reproductive health (13, 14) and also identified as EDC.

Several previous studies have reported the association of phthalates and heavy metals with uterine leiomyomata and endometriosis (15–17). The association of soy-food consumption with uterine leiomyomata was also reported (18, 19). However, these studies have focused primarily on the effect of a single chemical, without taking chemical interactions and confounding effects of other chemicals into consideration. The general population is exposed to these chemicals simultaneously and the real exposure pattern is mixture exposure. The additive effect or chemical interaction could exacerbate adverse effects on human health (20). Moving beyond the traditional approach of testing one chemical at one time to assessing the combined effects of chemical mixtures on outcomes is the key point of future epidemiology studies (21). The studies considering the effects of mixture exposure of EDCs on uterine leiomyomata and endometriosis are warranted.

By using the female population data from the National Health and Nutrition Examination Survey (NHANES) from 2001 to 2006, the study was conducted to examine the individual and combined associations between EDCs (here are phthalate, heavy metals, and equol) and uterine leiomyomata and endometriosis. Ten chemicals including monobutyl phthalate (MBP), monoethyl phthalate (MEP), mono(2-ethylhexyl) phthalate (MEHP), monobenzyl phthalate (MBzP), mono(3-carboxypropyl) phthalate (MCPP), mono-isobutyl phthalate (MIBP), cadmium (Cd), lead (Pb), mercury (Hg) and equol were analyzed in our study. Multivariable logistic regression model, weighted quantile sum (WQS) regression, and Bayesian kernel machine regression (BKMR) models were employed to more comprehensively explore the combined effects of mixed chemical exposure on uterine leiomyomata and endometriosis. Subanalyses were conducted by excluding the patients diagnosed with uterine leiomyomata and endometriosis for more than ten years, respectively. To reduce the potential bias induced by menopausal status, another subanalysis was conducted by restricting the participants to premenopause. Our results are expected to provide some fresh thinking in understanding the relationship between chemical mixture exposure and uterine leiomyomata and endometriosis.

## Methods

### Study Population

Data was extracted from NHANES, which used a multiple-layered probability sampling design focusing on a representative sample population located in counties across the US. The study samples were randomly divided into subgroups in each NHANES cycle and each subgroup had different chemicals measured (22). To maximize the sample size and the classes of EDCs included in our analysis, participants in NHANES from 2001 to 2006 with phthalate metabolites, equol, and heavy metal measured were candidates of our study. There were 4953 women aged between 20 and 54 years old who participated in NHANES 2001-2006. Women without complete measurement of urinary phthalate metabolites, equol, and whole blood heavy metals concentrations (n=1427 included) were excluded from further analysis. Those without answers to questions about uterine leiomyomata and endometriosis (n=1279 included) were excluded as well. Women without information of key covariates (including urinary creatinine levels, menarche age, body mass index (BMI), ovary removal, and usage of female hormone) were further excluded and 1204 samples were retained for the following analysis. This study was determined exempt from review by Nanjing Maternity and Child Health Hospital Institutional Review Board.

### Assessment of Chemical Exposures

Specimens, such as urine and whole blood, were obtained in mobile examination centers (MECs) and stored frozen at or below -20°C until analyzed. Our targeted chemicals were urinary phthalate metabolites, equol, as well as whole blood heavy metals. Analyses for phthalate metabolites were done by high-performance liquid chromatography-electrospray ionization-tandem mass spectrometry (HPLC-ESI-MS/MS) and equol by high performance liquid chromatography–atmospheric pressure photoionization–tandem mass spectrometry (HPLC–APPI–MS/MS). Blood Pb and Cd levels were measured using inductively coupled plasma mass spectrometry (ICP-MS). Total Hg concentrations in whole blood were measured by cold vapor atomic absorption spectrophotometry (CVAAS) (23). Twelve phthalate metabolites, three heavy metals, and equol were measured in all three NHANES cycles. Among the 16 chemicals, mono-cyclohexyl phthalate (MCHP), mono-isononyl phthalate (MNP), mono-n-octyl phthalate (MOP), and mono-n-methyl phthalate (MNM) were detected in less than 60% of the samples and excluded in the following analysis (**Table S1**). In addition, mono-(2-ethyl)-hexyl phthalate (MEHP), mono-(2-ethyl-5-hydroxyhexyl) phthalate (MEHHP), and mono-(2-ethyl-5-oxohexyl) phthalate (MEOHP) were the metabolites of diethylhexyl phthalate (DEHP) and MEHP, the most representative metabolite of DEHP, was included to represent the exposure levels of DEHP. Ten chemicals including MBP, MEP, MEHP, MBzP, MCPP, MIBP, Cd, Pb, Hg, and equol were retained in the following analysis. Enzymatic Roche Cobas 6000 analyzer was used to measure urinary creatinine concentration in grams per liter. The concentration of urinary creatinine was adjusted as a covariate to account for urinary dilution according to the recommendation (24).

### Assessment of Uterine Leiomyomata and Endometriosis

Regarding the diagnosis of uterine leiomyomata and endometriosis, two questions were asked: 1) “Has a doctor or other health professional ever told you that you had endometriosis?” 2) “Has a doctor or other health professional ever told you that you had uterine fibroids?” The participants who had positive answers were classified as patients and their age at first diagnosis was further inquired.

### Assessment of Covariates

Data on ovary removal and usage of female hormones was obtained from the NHANES reproductive health questionnaire. Information such as age, age of menarche, educational levels, and race/ethnicity were extracted from the home interview survey. BMI was obtained by body measurement. Current pregnant status was classified based on a reproductive health questionnaire and assays of urinary human chorionic gonadotropin (HCG). The information on menopausal status was extracted from the reproductive health questionnaire as previously described (25).

### Statistical Analysis

The t-test was used to compare group differences of continuous variables, which are shown as the mean and standard deviation (SD). While categorical variables are presented in the form of frequency and percentage, and the chi-square (χ2) test was used to compare differences between groups. Differences between groups were adjusted as covariates in the following analysis. Since the distributions of the concentrations of these ten chemicals were right-skewed, the exposure levels were ln-transformed to improve a normal distribution. The *Pearson* correlation was used to calculate the correlation coefficients among these chemicals.

Multivariable logistic regression, weighted quantile sum (WQS) regression, and Bayesian kernel machine regression (BKMR) were combined to comprehensively analyze the association between the targeted chemicals and uterine leiomyomata as well as endometriosis. The multivariable logistic regression model was used to estimate the odds ratio (ORs) adjusted for potential confounders, including age, ethnicity, pregnant status, ovary removal, usage of female hormones, BMI, menopause status, and urinary creatinine levels.

WQS and BKMR were conducted here to analyze the effects of mixed chemical exposure. A detailed description of these models could be found in our previous study (26). Briefly, a WQS index was calculated to represent the whole body burden of the targeted chemicals and its association with outcomes (uterine leiomyomata and endometriosis in our study) represents the association between chemical mixture exposure and health outcomes. The WQS index is a combination of all the targeted chemicals and the weights of each chemical in the index indicate their contribution to the whole effect. For WQS regression, the positive and negative effects on outcomes were estimated separately with bootstrapping 10,000 times, and chemicals were scored as 0, 1, and 2 to represent the 1^st^, 2^nd^, and 3^rd^ tertile exposure levels, respectively. To estimate the overall association of the chemical mixture with outcomes, the BKMR model with a hierarchical variable selection method with 50,000 iterations was also employed. MBP, MEP, MEHP, MBzP, MCPP, and MBP were grouped into group 1, equol into group 2, and Cd, Pb, and Hg into group 3, based on their similar sources and high correlations with each other. The joint effect was calculated by comparing all the chemicals exposed at their 60^th^ percentile levels or above with all of them at their 50^th^ percentile. The group probabilities of inclusion (groupPIP) and conditional PIP (condPIP) indicate the probability of the group and the chemical in each group included in the model and represent their contribution to the overall association. By fixing all the other chemicals at their median exposure levels, the dose-response relationship was also estimated in the BKMR model. Interactions between each two of the chemicals when fixing other chemicals at their 50^th^ percentile exposure levels were also explored.

In parallel with our main analyses, patients diagnosed with uterine leiomyomata (N=1152 included) or endometriosis (N=1173 included) more than ten years were also excluded to minimize the bias induced by exposure level changes after disease diagnosis. In another subgroup analysis, participants were restricted to premenopause (N=1045) to reduce the influence of exposure level changes after postmenopause.

Previous studies find when the primary variables used to calculate the sampling weights are already included as covariates, the weighted estimation could introduce over-adjusted bias (27, 28). Besides, whether the sampling weight is appropriate to apply to complex statistic models like WQS and BKMR is not clear. Population stratification weights were not used in this study. All the analyses were performed with R software (R 3.6.0). “gWQS” and “BKMR” packages were used to conduct WQS and BKMR regression. The R code of this study could be found in **Supplementary Material**. A p-value < 0.05 was considered to be statistically significant.

## Results

### Study Population Characteristics

In total, there were 1204 participants included in our study, including 151 (12.5%) with uterine leiomyomata and 77 (6.4%) with endometriosis. **Table 1** shows the characteristics of the analyzed population. There were significant differences between diseased and non-diseased participants in the field of age, ethnicity, ovary removal, the usage of female hormones, and menopause status. Women with uterine leiomyomata or endometriosis had older age and a higher proportion of ovary removal history, the usage of female hormones, and the postmenopause status. Besides, uterine leiomyomata patients had a higher proportion of non-pregnant status and obesity.

**Table 1:** Baseline characteristics of the analyzed participants (N=1204).

Characteristics	Non-endometriosis	Endometriosis	*P* value	Non-uterine fibroid	Uterine fibroid	*P* value
	n = 1127	n = 77		n = 1053	n = 151	
**Age, year**	34.69 (9.68)	38.30 (8.82)	0.001	33.64 (9.34)	43.83 (6.77)	<0.001
**Menarche age**	12.61 (1.77)	12.29 (1.93)	0.238	12.61 (1.76)	12.38 (1.87)	0.138
**Ethnicity**			0.033			<0.001
Non-Hispanic white	357 (31.7)	16 (20.8)		346 (32.9)	27 (17.9)	
Non-Hispanic black	532 (47.2)	48 (62.3)		517 (49.1)	63 (41.7)	
Hispanic/other	238 (21.1)	13 (16.9)		190 (18.0)	61 (40.4)	
**Education level**			0.631			0.639
lower than high school	247 (21.9)	12 (15.6)		232 (22.0)	27 (17.9)	
high school	238 (21.1)	18 (23.4)		224 (21.3)	32 (21.2)	
Some College or AA degree	384 (34.1)	28 (36.4)		355 (33.7)	57 (37.7)	
College Graduate or above	258 (22.9)	19 (24.7)		242 (23.0)	35 (23.2)	
**BMI group**			0.365			0.001
Non-obesity	718 (63.7)	53 (68.8)		692 (65.7)	79 (52.3)	
Obesity	409 (36.3)	24 (31.2)		361 (34.3)	72 (47.7)	
**Current pregnancy status**			0.252			<0.001
No	906 (80.4)	66 (85.7)		828 (78.6)	144 (95.4)	
Yes	221 (19.6)	11 (14.3)		225 (21.4)	7 (4.6)	
**Ovary removed**			<0.001			<0.001
No	1069 (94.9)	54 (70.1)		1019 (96.8)	104 (68.9)	
Yes	58 (5.1)	23 (29.9)		34 (3.2)	47 (31.1)	
**Female hormone**			<0.001			<0.001
No	1027 (91.1)	55 (71.4)		975 (92.6)	107 (70.9)	
Yes	100 (8.9)	22 (28.6)		78 (7.4)	44 (29.1)	
**Menopause status**			<0.001			<0.001
Premenopause	993 (88.1)	52 (67.5)		960 (91.2)	85 (56.3)	
Postmenopause	134 (11.9)	25 (32.5)		93 (8.8)	66 (43.7)	

BMI, body mass index. Data are presented as mean ± SD or n (%). The t-test and χ2 test were between the endometriosis and no endometriosis groups or between the uterine leiomyomata and no uterine leiomyomata groups.

### Concentrations of Urinary Phthalates Metabolites, Equol, and Whole Blood Heavy Metals and Their Correlation

The distribution of the ten chemicals is shown in **Table S1**. Their detection frequency range from 63.3% (Cd) to 99.5% (MEP). Among the phthalate metabolites, MEP, the metabolite of diethyl phthalate (DEP), had the highest concentration. The concentrations of the phthalate metabolites were significantly positively associated with each other (**Figure S1**, range from 0.21 to 0.69, P<0.05). Equol also had a mild positive association with phthalate metabolites (range from 0.09 to 0.25, P<0.05). Among the heavy metals, a moderate positive association was found between Cd and Pb (r=0.37, P<0.05). The correlations between phthalate metabolites and heavy metals were weak.

### Classical Single Chemical Analysis to Assess the Association Between Chemical Exposure and Uterine Leiomyomata and Endometriosis

**Figure 1:**
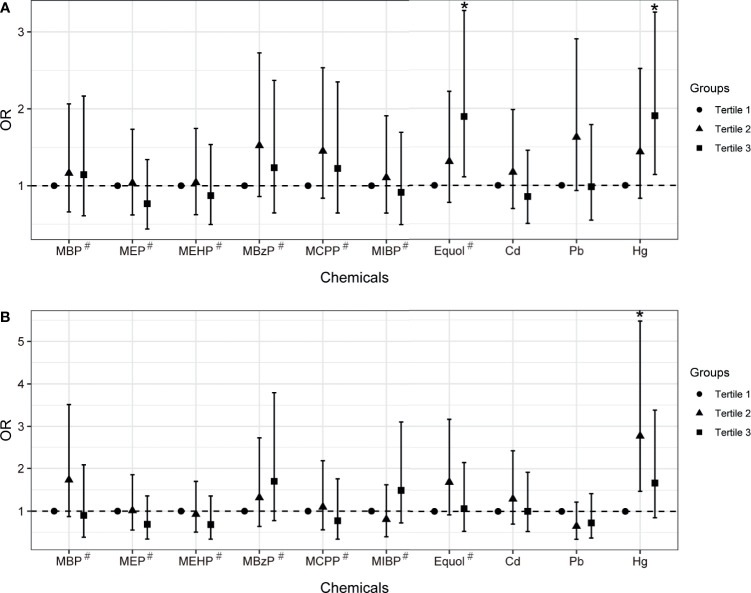
The associations between chemical exposure and uterine leiomyomata **(A)** or endometriosis **(B)** in women aged between 20 and 54 years old with multivariable logistic regression model. Models were adjusted for age, ethnicity, BMI group, ovary removal, female hormone usage, pregnant status, and menopause status. Models^#^ were further adjusted for log-transformed urinary creatinine levels. *Indicates P-value < 0.05.

The multivariable logistic regression model was firstly employed to assess the association between single chemical/metabolite concentration and uterine leiomyomata and endometriosis (**Figure 1**, **Table S2**). After adjusting for covariates that were different between groups, equol was found significantly associated with uterine leiomyomata (OR: 1.23, 95% CI: 1.07-1.41, FDR P<0.05). When further modeling exposure variables into tertile groups, equol (OR: 1.90, 95% CI: 1.11-3.27, P for trend =0.019) and Hg (OR: 1.91, 95% CI: 1.14-3.25, P for trend =0.014) had statistically positive associations with uterine leiomyomata when comparing the third tertile with the first one. Hg exposure was significantly associated with endometriosis in the second tertile (OR: 2.77, 95% CI: 1.47-5.47).

### WQS and BKMR Models to Assess the Combined Association Between Multiple Exposures and Uterine Leiomyomata and Endometriosis

**Figure 2:**
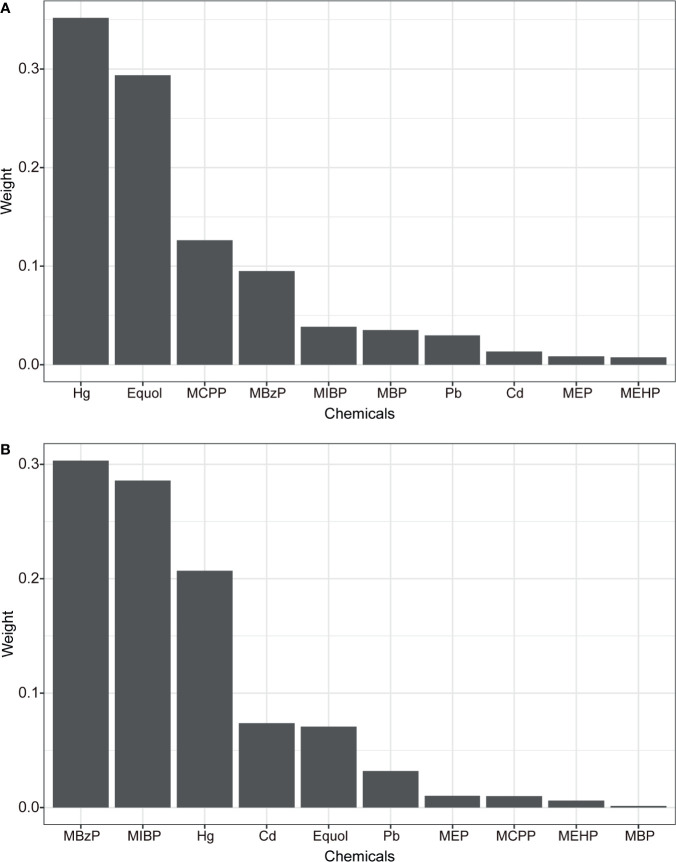
The weights of each chemical in positive WQS model regression index for uterine leiomyomata **(A)** and endometriosis **(B)**. Models were adjusted for age, ethnicity, BMI group, ovary removal, female hormone usage, pregnant status, menopause status, and log-transformed urinary creatinine levels.

The WQS and BKMR models were employed to estimate the combined effect of chemical/metabolites mixture exposure on uterine leiomyomata and endometriosis. In the WQS model (**Table 2**), mixed exposure of chemicals had a significant positive association with uterine leiomyomata (OR: 2.54 95% CI: 1.52, 4.29) but a marginal association with endometriosis (OR: 2.01, 95% CI: 0.98, 4.15). This suggests the whole chemical body burden had a positive association with uterine leiomyomata and the distribution of the weights showed Hg and equol had the highest weights (weight=0.35 and 0.29, respectively) and made the biggest contribution while MEHP had the lowest weight (**Figure 2A**). In the marginal positive association with endometriosis, MBzP and MIBP had the highest weights (**Figure 2B**) and MEHP and MBP weighted low. Exploring the negative associations between WQS index and uterine leiomyomata or endometriosis, no significant associations were found (**Table 2**).

**Table 2:** WQS model to estimate associations between WQS index and uterine leiomyomata and endometriosis.

Outcomes	OR (95% CI)	P value
b1_positive		
Uterine leiomyomata	2.54 (1.52, 4.29)	<0.001
Endometriosis	2.01 (0.98, 4.15)	0.058
b1_negative		
Uterine leiomyomata	0.70 (0.39, 1.24)	0.222
Endometriosis	0.51 (0.25, 1.05)	0.067

OR, odds ratio; CI, confidence interval. OR estimation represents the odds ratios of uterine leiomyomata or endometriosis as one tertile increased in the WQS index. The positive and negative association was estimated respectively. Models were adjusted for age, ethnicity, BMI group, ovary removal, female hormone usage, pregnant status, menopause status, and log-transformed urinary creatinine levels.

**Figure 3:**
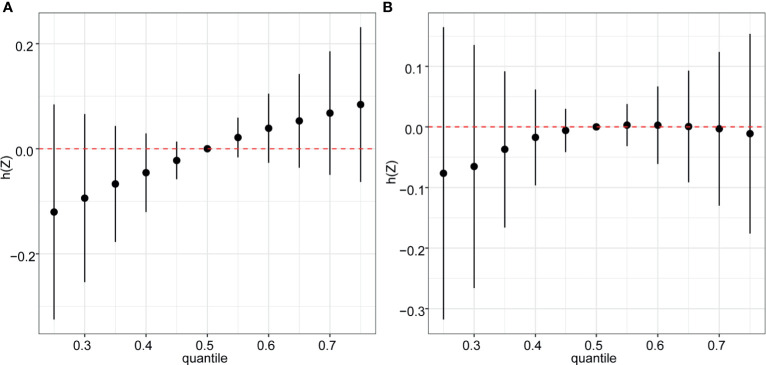
Overall risk (95% CI) of the mixture on uterine leiomyomata **(A)** and endometriosis **(B)** when comparing all the chemicals at different percentiles with all of them fixed at the median level. Models were adjusted for age, ethnicity, BMI group, ovary removal, female hormone usage, pregnant status, menopause status, and log-transformed urinary creatinine levels.

**Figure 4:**
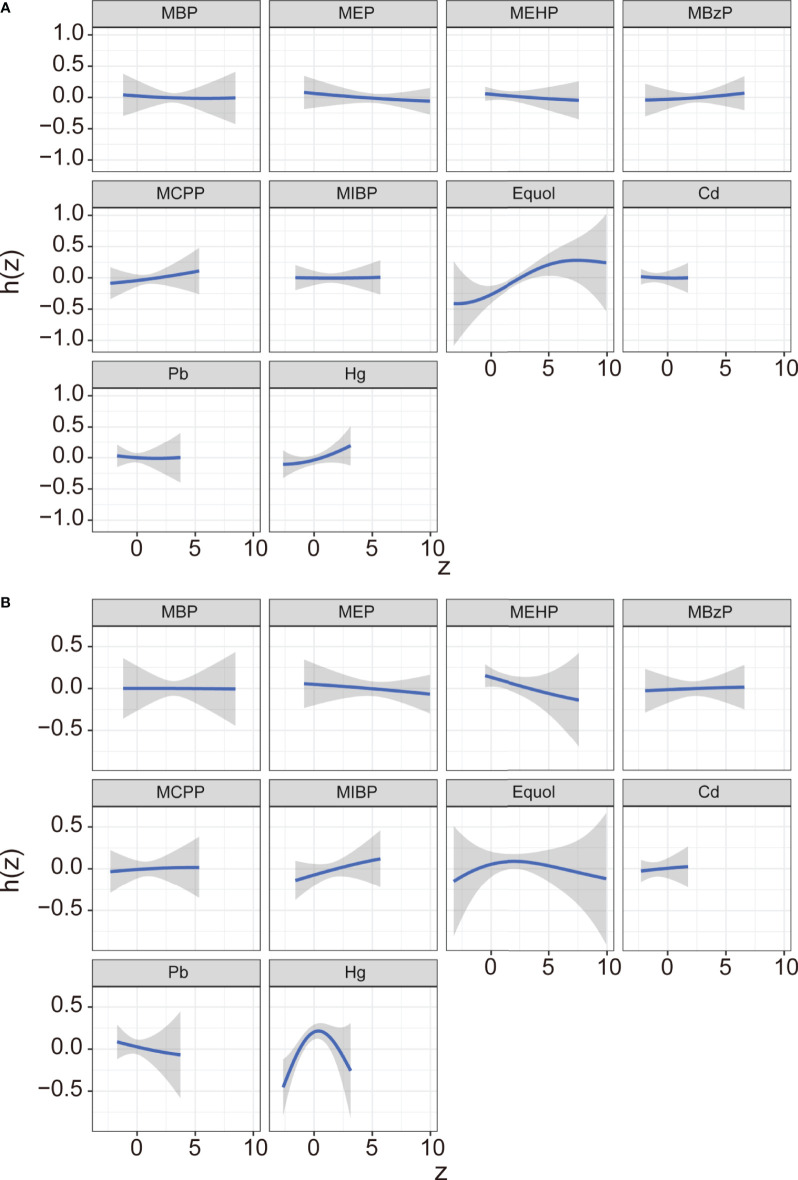
Exposure-response function (95% CI) between selected chemical exposure and uterine leiomyomata **(A)** or and endometriosis **(B)** with fixing all the other chemicals at their median level. Models were adjusted for age, ethnicity, BMI group, ovary removal, female hormone usage, pregnant status, menopause status, and log-transformed urinary creatinine levels.

In the BKMR model, ten chemicals were grouped into three groups according to their similar exposure sources and correlations. Firstly, the overall associations between the mixed chemical exposure and the outcomes were estimated. When all the chemicals were at their 60^th^ percentile or higher compared to their median exposure levels, a positive tendency was found between mixture exposure and uterine leiomyomata (**Figure 3A**) while the reverse U shape association was found between mixture exposure and endometriosis (**Figure 3B**). The contribution of chemicals in the overall effect and their dose-response curve were further explored. The probabilities of inclusion (PIPs) derived from the BKMR model in uterine leiomyomata and endometriosis regression for the groups (groupPIP) and each chemical (condPIP) are summarized in **Table S3**. In the uterine leiomyomata model, the groupPIP of the equol group was 0.65, while the groupPIPs of metals and phthalate metabolites were 0.43 and 0.23, respectively. The PIP of equol (groupPIP=0.65) was extremely high, indicating that the equol was the most influential for probable uterine leiomyomata. In the metals group, Hg made the most contribution (CondPIP: 0.55), while MCPP drove the main effect in the phthalates group (CondPIP = 0.38). In the endometriosis analysis, the metal group has the highest groupPIP (groupPIP=0.76) with Hg made the highest CondPIP (CondPIP: 0.79). MEHP and MIBP had the highest condPIP in the phthalate metabolite group (groupPIP: 0.36, MEHP condPIP: 0.36, MIBP condPIP: 0.26). The trends of exposure-response functions of the 10 chemicals were further explored. With all the other chemicals fixed at their 50^th^ exposure levels, equol and Hg showed increasing associations with uterine leiomyomata (**Figure 4A**), whereas MEHP showed an inverse association. No evidence of chemical interaction was found among each two of these chemicals (**Figure S2**). For the dose-response curve of chemical exposure and endometriosis, MIBP showed a positive trend and MEHP showed a negative trend. Hg exposure showed a reverse U shape with endometriosis (**Figure 4B**). Besides, Hg had interaction with equol and MEHP in their association with endometriosis in BKMR bivariable analysis (**Figure S3**).

### Subanalysis Based on the Diagnosis Duration and Menopause Status

Due to the cross-sectional study design, the chemical exposure levels could have changed after diagnosis and induce exposure bias in this study. Firstly, the correlation between chemical exposure levels and the number of years after uterine leiomyomata and endometriosis diagnosis was analyzed (**Figure S4**). The results show that only Pb was significantly correlated with the number of years since uterine leiomyomata diagnosis (**Figure S4A**, r=0.309, P=0.001) and Hg with the years after endometriosis diagnosis (**Figure S4B**, r=0.269, P=0.019). Secondly, women diagnosed with uterine leiomyomata or endometriosis more than 10 years before NHANES examination (52 uterine leiomyomata patients and 31 endometriosis patients excluded) were excluded to further reduce the exposure bias. In multivariate logistic regression, the ORs for women with uterine leiomyomata in the 3^rd^ tertile of equol and Hg exposure levels versus the lowest tertiles were 1.89 (95% CI: 1.03-3.50, P for trend=0.034) and 1.85 (95% CI: 1.05-3.35, P for trend=0.033), respectively (**Table S4**). Besides, Cd and Hg exposure in the second tertile were significantly associated with endometriosis (OR: 2.35, 95% CI: 1.11-5.32; OR: 2.15, 95% CI: 1.06-4.57). The combined association of chemical mixture exposure was positively associated with uterine leiomyomata in the WQS model (OR: 2.52, 95% CI: 1.35, 4.76) (**Table S5**) and equol and Hg made the most contribution to the positive WQS index and MEHP made the lowest (**Figure S5**). Neither significant positive nor negative associations were found between combined chemical exposure and endometriosis (**Table S5**). A positive trend association was found between mixed chemical exposure and uterine leiomyomata in the BKMR model (**Figure S6A**). Besides, the positive trend of dose-response association was also found in equol and Hg exposure and uterine leiomyomata (**Figure S7A**). Similar to the main results, an inverted-U shape trend was found between chemical mixture exposure and endometriosis (**Figure S6B**) with MIBP positively, MEHP negatively, and Hg inverted-U-shape associated with endometriosis (**Figure S7B**).

The menopause status could play a role in influencing human metabolite status and further change the way chemicals are exposed in the human body. In the following analyses, participants were restricted to premenopause and the main analyses were repeated. In classic single chemical analysis, no single chemical exposure was associated with uterine leiomyomata while MBzP was found significantly positively associated with endometriosis (OR: 2.68, 95% CI: 1.05-7.23, P for trend=0.055) (**Table S6**). WQS regression model was also employed and the whole-body burden of chemical exposure was found significantly associated with both uterine leiomyomata (OR: 2.34, 95% CI: 1.22, 4.57) and endometriosis (OR: 2.18, 95% CI: 1.03, 4.70) (**Table 3**). Hg and equol weighted highest in WQS model of uterine leiomyomata, and MBzP and MIBP weighted highest in the model of endometriosis (**Figure S8**). In the BKMR model, similar results were found to the main results (**Figures S9, S10**).

**Table 3:** WQS model to estimate the association between WQS index and uterine leiomyomata and endometriosis in premenopausal participants (N=1045).

Outcomes	OR (95% CI)	P value
b1_positive		
Uterine leiomyomata	2.34 (1.22, 4.57)	0.011
Endometriosis	2.18 (1.03, 4.70)	0.043
b1_negative		
Uterine leiomyomata	0.64 (0.33, 1.22)	0.177
Endometriosis	0.46 (0.19, 1.09)	0.079

OR, odds ratio; CI, confidence interval. OR estimation represents the odds ratios of uterine leiomyomata or endometriosis as one tertile increased in the WQS index. The positive and negative association was estimated respectively. Models were adjusted for age, ethnicity, BMI group, ovary removal, female hormone usage, pregnant status, and log-transformed urinary creatinine levels.

## Discussion

Our study combined the routine method (multivariable logistic regression) and advanced approaches (WQS and BKMR) to estimating the joint effects of a mixture to comprehensively evaluate the association of ten endocrine-related chemicals and uterine leiomyomata as well as endometriosis in the United States population. In logistic regression, equol and Hg were positively associated with uterine leiomyomata. The positive combined association of these chemicals with uterine leiomyomata was identified and the negative association of MEHP with uterine leiomyomata was also indicated in WQS and BKMR models. No association was found between mixture exposure and endometriosis. The results were consistent when we further excluded patients diagnosed more than ten years. In a subanalysis with only premenopausal participants included, the significant positive association of chemical mixture exposure and endometriosis were further identified with MIBP and MBzP made the most contribution and MEHP made the lowest. We stressed the importance of fully evaluating the relationships between EDCs mixtures and gynecological diseases.

To comparing with previous studies, the association of phthalate metabolites, heavy metals, and equol exposure with uterine leiomyomata and endometriosis was firstly assessed in classic multivariable logistic regression models. Equol and Hg were found to be positively associated with uterine leiomyomata in our study. In another cross-sectional NHANES study, which assessed heavy metal exposure association with uterine leiomyomata, Hg level in women with uterine leiomyomata was significantly higher compared with women without uterine leiomyomata (29). Their relationship could be interpreted by several studies *in vitro*, which illustrated that mercury has the estrogen-like effect to induce leiomyoma cell proliferation and stimulate tumor growth (30, 31). However, another cross-sectional study found no positive association between Hg and uterine leiomyomata (32). Although Hg exposure level was not correlated with the number of years after uterine leiomyomata diagnosis and excluding patients diagnosed with uterine leiomyomata for more than ten years didn’t change the main results, heavy metals like Hg tend to accumulate in the human body and could lead to exposure bias. Prospective studies and cross-sectional studies with uterine leiomyomata newly developed are warranted. Other possible reasons for the discrepancy might come from the different covariates considered and association distortion induced by the potential multicollinearity with other chemicals (29, 33).

Equol, the metabolites of soy isoflavone daidzein, was found positively associated with uterine leiomyomata in our study. As the estrogenic effect of equol, it has been recommended to supply the estrogens that are lost at menopause for reducing menopause symptoms and preventing weak bones. But also due to its estrogen-like effects, its potential to exacerbate estrogen-dependent issues like leiomyomata should be considered. Our study found a positive association between urinary equol levels and uterine leiomyomata, which hasn’t been reported before. Studies reported soy-based formulation during infancy and frequency milk and soybean consumption during adult increase the risk of uterine leiomyomata (19, 34). An animal study found equol had no major abnormality on the ovary but triggered hyperplasia of uterine tissue by increasing luminal epithelial cell height, and myometrial and stromal thickness, which effect is prolonged and could further lead to uterine leiomyomata (35). There was insufficient evidence to determine its safety usage duration and doses in women. The potential health risk of soy food consumption in fertile women and postmenopausal women should be further evaluated.

In analyses of the phthalate metabolites, the evidence of the low weight in positive WQS indexes and the negative dose-response trend between MEHP exposure and uterine leiomyomata and endometriosis showed MEHP negatively associated with uterine leiomyomata as well as endometriosis. Our results agree with a cross-sectional study that MEHP was negatively associated with the combined condition of uterine leiomyomata and endometriosis (4). Other studies also found the inverse associations between MEHP exposure and risk of uterine leiomyomata (36, 37). Furthermore, *in vivo* and *in vitro* studies indicate that there is a growing tendency among MEHP to stop ovulating or delay ovulation, decrease the synthesis of estradiol, and decrease serum progesterone levels (38, 39). Our finding also suggests that MBzP and MIBP made the most contribution in the positive association between the chemical mixture and endometriosis in premenopausal participants, which association has also been suggested in other studies (37, 40). However, the phthalate metabolites have short half-lives and usually do not bioaccumulate in human body. Although some evidence suggests reproductive-aged women are somehow consistently exposed to phthalate and the urinary concentrations of phthalate metabolites are little variate during menstrual cycle (4) and our result found no correlation between phthalate metabolite exposure levels and the age after uterine leiomyomata or endometriosis diagnosis, it still needs to be cautious in interpreting our results.

Given that chemicals were exposed simultaneously and commonly exposure sources and metabolism pathways could induce high colinearity, the effect of a chemical on health outcomes could be concealed or exaggerated due to other related chemicals. More researchers call for the exploration of health effects on mixed chemical exposure (41, 42). WQS and BKMR, two advanced models, were employed to estimate the whole effect of chemical mixtures and each chemical’s contribution by considering their correlations. The positive associations of equol and Hg with uterine leiomyomata in logistic regression were confirmed in WQS and BKMR models. Also, in the subanalysis with premenopausal participants, none of the single chemicals was associated with uterine leiomyomata but WQS model identified their significant positive association, which is consistent with the findings that WQS is more sensitive than the classic linear regression model (43, 44). Besides, MEHP had a negligible weight in the positive association of WQS index with uterine leiomyomata and endometriosis (**Figure 2**), which indicates MEHP had a null or negative association with the diseases. These results were supported by the negative dose-response curve of MEHP with uterine leiomyomata and endometriosis in the BKMR model, which experts in the identification of non-linear multivariable exposure-response functions (45). In our analysis, Hg was found to interact with other chemicals in the dose-response relationship with endometriosis (**Figure S3**). For example, equol had a positive dose-response curve with endometriosis when Hg was exposed at low levels and other chemicals at their median levels (**Figure S3**). However, when Hg was exposed at its median or high exposure levels, an inverse-U trend was found between equol and endometriosis. In epidemiology studies, the relationship of a single chemical with outcome should be fully evaluated by considering other chemicals’ interaction and BKMR is expert in answering this research question. WQS and BKMR models we used here also have some limitations. By applying the WQS model, the chemicals are assumed to have null or same direction associations with outcomes and the associations are linear and additive. A novel Bayesian extension of the WQS regression (BWQS) was developed without restriction on the effect direction, having the ability to estimate 95% Credible Interval of the chemical weights and having higher statistic power (46). Another alternative model, quantile g-computation, is a causal inference method, which doesn’t assume directional homogeneity and allows for nonlinearity and nonadditivity of the chemical effect (47). These new methods could provide more evidence in future studies. For the BKMR model, the overall risk is estimated with all the chemicals at different percentile compared with their exposure levels at the median. But the real exposure pattern is a combination of chemicals with different exposure levels. The results of WQS and BKMR analysis should be interpreted cautiously with considering their limitations.

Our study employed multiple statistical models to comprehensively explore the associations of chemical mixture with gynecological disorders. Some limitations should be noted in our study. Firstly, the NHANES survey is cross-sectionally designed. The chemical exposure was measured years later after the diagnosis of the disease. The changes in exposure levels after diagnosis could induce measurement bias. Subanalyses were carried out by excluding women diagnosed with uterine leiomyomata and endometriosis more than ten years before chemical measurement, respectively. No major changes were found in the results, which to some extent indicate their associations are stable. The second limitation of the study is the definition of outcomes. The self-reported disease could underestimate the prevalence especially for uterine leiomyomata and endometriosis as a large proportion of patients have no symptoms (48). The simple substitution of chemical concentration below the limit of detection should also be noted in our study for potential measurement bias. Other EDCs such as pesticides, phenols, and polychlorobiphenyls were not included in our analysis as the exposure data was not available in the subsamples we chose. Prospective cohort studies with precise endpoints diagnosis and more related chemicals included are warranted.

## Conclusion

Multivariable logistic regression model, WQS regression, and BKMR regression models have arisen to analyze the joint effects of ten chemicals on the overall risk of uterine leiomyomata and endometriosis. Equol and Hg were the most significant chemicals associated with uterine leiomyomata. In subanalysis with excluding patients diagnosed more than ten years, the results were consistent. In a subanalysis only included premenopausal participants, the positive association between the chemical mixture and endometriosis was also identified and MIBP and MBzP made the most contribution. MEHP was found negatively associated with uterine leiomyomata and endometriosis by combining the results of WQS and BKMR models. This study provides new evidence that endocrine-related chemical mixture is closely related to female reproduction health and further research is needed to explore the detailed mechanism.

## Data Availability Statement

The raw data supporting the conclusions of this article will be made available by the authors, without undue reservation.

## Author Contributions

YZ, data curation, formal analysis, writing - original draft, writing - review and editing. YL, data curation, writing - original draft, writing - review and editing. HM, writing - review and editing. QX, formal analysis, funding acquisition, supervision, writing - review and editing. XW, funding acquisition, project administration, supervision, writing - review and editing. All authors contributed to the article and approved the submitted version.

## Funding

This work was supported by grants from the National Natural Science Foundation of China (grant no. 81801413), Nanjing Health Science and Technology Development Project (grant no. ZKX18045), Key Discipline of Maternal and Child Health in Jiangsu Province (grant no. FXK201756), and the Technology Development Foundation of Nanjing Medical University, China (grant no. 2017NJMUZD071).

## Conflict of Interest

The authors declare that the research was conducted in the absence of any commercial or financial relationships that could be construed as a potential conflict of interest.

## Publisher’s Note

All claims expressed in this article are solely those of the authors and do not necessarily represent those of their affiliated organizations, or those of the publisher, the editors and the reviewers. Any product that may be evaluated in this article, or claim that may be made by its manufacturer, is not guaranteed or endorsed by the publisher.

## Abbreviations

BKMR, Bayesian kernel machine regression; Cd, cadmium; CI, confidence interval; DF, Detection frequency; EM, endometriosis; Hg, mercury; LOD, lower limit of detection; MBP, monobutyl phthalate; MBzP, monobenzyl phthalate; MEP, monoethyl phthalate; MEHP, mono(2-ethylhexyl) phthalate; MIBP, mono-isobutyl phthalate; MCPP, mono(3-carboxypropyl) phthalate; NHANES, National Health and Nutrition Examination Survey; OR, odds ratio; Pb, lead; PIP, probabilities of inclusion; UL, uterine leiomyomata; WQS, weighted quantile sum.
