# Supplementary material for: Combined Exposure to Multiple Endocrine Disruptors and Uterine Leiomyomata and Endometriosis in US Women
Source: Front Endocrinol (Lausanne). 2021 Aug 20;12:726876. doi: 10.3389/fendo.2021.726876 (PMC8418539; doi:10.3389/fendo.2021.726876)
Supplement: Supplementary file 1 [file DataSheet_1.docx]

Supplementary Material

**Table S1**. Distribution of the chemicals (N=1204), NHANES, 2001–2006

| Chemicals | DF (%) | GM | Mean | Percentile | | | | |
| --- | --- | --- | --- | --- | --- | --- | --- | --- |
|  |  |  |  | 5 | 25 | 50 | 75 | 95 |
| MBP (ng/mL) | 98.7 | 20.89 | 41.60 | 2.50 | 10.50 | 23.00 | 43.20 | 121.70 |
| MCHP (ng/mL) | 7.1 | 0.45 | 0.51 | 0.43 | 0.43 | 0.43 | 0.43 | 0.80 |
| MEP (ng/mL) | 99.5 | 160.27 | 527.34 | 15.08 | 58.34 | 153.71 | 400.88 | 2094.69 |
| MEHP (ng/mL) | 71.8 | 3.55 | 12.16 | 0.85 | 0.85 | 3.20 | 8.10 | 41.38 |
| MNP (ng/mL) | 6.2 | 1.19 | 1.66 | 1.09 | 1.09 | 1.09 | 1.09 | 1.85 |
| MOP (ng/mL) | 1.0 | 1.32 | 1.32 | 1.31 | 1.31 | 1.31 | 1.31 | 1.31 |
| MB_Z_P (ng/mL) | 98.8 | 8.80 | 21.25 | 0.81 | 3.89 | 9.90 | 20.86 | 72.32 |
| MNM (ng/mL) | 49.8 | 1.65 | 3.84 | 0.78 | 0.78 | 0.78 | 3.00 | 11.28 |
| MCPP (ng/mL) | 92.2 | 2.16 | 4.27 | 0.30 | 1.10 | 2.20 | 4.38 | 11.68 |
| MEHHP (ng/mL) | 98.2 | 20.06 | 67.12 | 2.40 | 8.43 | 19.15 | 44.05 | 226.33 |
| MEOHP (ng/mL) | 96.6 | 14.03 | 44.66 | 1.60 | 5.73 | 13.35 | 30.80 | 151.13 |
| MIBP (ng/mL) | 84.7 | 4.13 | 8.08 | 0.70 | 1.90 | 4.35 | 9.20 | 25.35 |
| Equol (ng/mL) | 72.1 | 8.79 | 88.08 | 2.33 | 2.33 | 7.93 | 16.78 | 64.40 |
| Cd (ug/L) | 63.3 | 0.39 | 0.53 | 0.20 | 0.20 | 0.30 | 0.57 | 1.60 |
| Pb (ug/L) | 98.0 | 1.00 | 1.26 | 0.39 | 0.70 | 0.96 | 1.41 | 3.09 |
| Hg (ug/L) | 96.3 | 0.89 | 1.39 | 0.20 | 0.47 | 0.90 | 1.61 | 4.38 |

DF: Detection frequency, GM: Geometric mean, MBP: monobutyl phthalate, MCHP: mono-cyclohexyl phthalate, MEP: monoethyl phthalate, MEHP: mono(2-ethylhexyl) phthalate, MNP: mono-isononyl phthalate, MOP: mono-n-octyl phthalate, MBzP: monobenzyl phthalate, MNM: mono-n-methyl phthalate, MCPP: mono(3-carboxypropyl) phthalate, MEHHP: mono-(2-ethyl-5-hydroxyhexyl) phthalate, MEOHP: mono-(2-ethyl-5-oxohexyl) phthalate, MIBP: mono-isobutyl phthalate, Cd: cadmium, Pb: lead, Hg: mercury

**Table S2.** The odds ratio for the association between single urinary/blood chemical exposure and uterine leiomyomata and endometriosis in women aged between 20 and 54 years old

| Exposure | Estimated OR (95% CI) by continuous exposure | |  | Estimated ORs (95% CI) by categorical exposure | | | | |
| --- | --- | --- | --- | --- | --- | --- | --- | --- |
|  |  |  |  |  |  |  |  |  |
|  | OR (95% CI) | FDR P |  | T1 | T2 |  | T3 | P for trend |
| Uterine leiomyomata | |  |  |  |  |  |  |  |
| MBP^#^ | 0.98 (0.77, 1.24) | 0.863 |  | 1.00 | 1.16 (0.66, 2.06) |  | 1.14 (0.61, 2.16) | 0.722 |
| MEP^#^ | 0.91 (0.77, 1.06) | 0.697 |  | 1.00 | 1.03 (0.62, 1.73) |  | 0.77 (0.44, 1.34) | 0.315 |
| MEHP^#^ | 0.94 (0.79, 1.11) | 0.793 |  | 1.00 | 1.04 (0.62, 1.74) |  | 0.87 (0.50, 1.54) | 0.613 |
| MBzP^#^ | 1.03 (0.83, 1.27) | 0.863 |  | 1.00 | 1.52 (0.86, 2.73) |  | 1.23 (0.65, 2.37) | 0.690 |
| MCPP^#^ | 1.14 (0.90, 1.45) | 0.697 |  | 1.00 | 1.45 (0.84, 2.53) |  | 1.22 (0.65, 2.35) | 0.626 |
| MIBP^#^ | 0.97 (0.77, 1.21) | 0.863 |  | 1.00 | 1.11 (0.64, 1.91) |  | 0.91 (0.49, 1.69) | 0.714 |
| Equol^#^ | 1.23 (1.07, 1.41) | 0.025 |  | 1.00 | 1.31 (0.78, 2.22) |  | 1.90 (1.11, 3.27) | 0.019 |
| Cd | 0.93 (0.71, 1.19) | 0.794 |  | 1.00 | 1.17 (0.70, 1.99) |  | 0.85 (0.50, 1.46) | 0.451 |
| Pb | 0.86 (0.60, 1.22) | 0.793 |  | 1.00 | 1.63 (0.93, 2.90) |  | 0.98 (0.55, 1.79) | 0.608 |
| Hg | 1.27 (1.03, 1.57) | 0.142 |  | 1.00 | 1.44 (0.83, 2.52) |  | 1.91 (1.14, 3.25) | 0.014 |
| Endometriosis | |  |  |  |  |  |  |  |
| MBP^#^ | 1.05 (0.79, 1.40) | 0.798 |  | 1.00 | 1.73 (0.87, 3.51) |  | 0.90 (0.39, 2.08) | 0.560 |
| MEP^#^ | 0.91 (0.75, 1.10) | 0.798 |  | 1.00 | 1.01 (0.55, 1.85) |  | 0.69 (0.35, 1.36) | 0.282 |
| MEHP^#^ | 0.84 (0.67, 1.03) | 0.598 |  | 1.00 | 0.93 (0.51, 1.70) |  | 0.68 (0.34, 1.35) | 0.279 |
| MBzP^#^ | 1.08 (0.84, 1.39) | 0.798 |  | 1.00 | 1.32 (0.64, 2.73) |  | 1.70 (0.78, 3.79) | 0.181 |
| MCPP^#^ | 1.07 (0.79, 1.44) | 0.798 |  | 1.00 | 1.10 (0.56, 2.18) |  | 0.77 (0.34, 1.76) | 0.476 |
| MIBP^#^ | 1.23 (0.95, 1.60) | 0.598 |  | 1.00 | 0.81 (0.40, 1.62) |  | 1.49 (0.72, 3.11) | 0.220 |
| Equol^#^ | 1.00 (0.84, 1.17) | 0.991 |  | 1.00 | 1.69 (0.92, 3.16) |  | 1.06 (0.53, 2.15) | 0.921 |
| Cd | 1.06 (0.78, 1.43) | 0.798 |  | 1.00 | 1.29 (0.70, 2.42) |  | 1.00 (0.53, 1.92) | 0.940 |
| Pb | 0.85 (0.54, 1.32) | 0.798 |  | 1.00 | 0.65 (0.34, 1.22) |  | 0.73 (0.37, 1.42) | 0.363 |
| Hg | 1.19 (0.92, 1.54) | 0.631 |  | 1.00 | 2.77 (1.47, 5.47) |  | 1.67 (0.85, 3.38) | 0.207 |

T: tertile; OR: odds ratio; CI: confidence interval. Models were adjusted for age, ethnicity, BMI group, ovary removal, female hormone usage, pregnant status, and menopause status. Models^#^ were further adjusted for log-transformed urinary creatinine levels. The association significance for continuous chemical exposure was presented as Benjamini & Hochberg corrected P values (FDR P).

**Table S3**. GroupPIP and condPIP in uterine leiomyomata and endometriosis model, using BKMR model in NHANES 2001–2006 (N = 1204)

| Chemicals | Group | Uterine leiomyomata | |  | Endometriosis | |
| --- | --- | --- | --- | --- | --- | --- |
|  |  | groupPIP | condPIP |  | groupPIP | condPIP |
| MBP | 1 | 0.23 | 0.14 |  | 0.36 | 0.10 |
| MEP | 1 | 0.23 | 0.09 |  | 0.36 | 0.08 |
| MEHP | 1 | 0.23 | 0.09 |  | 0.36 | 0.36 |
| MBzP | 1 | 0.23 | 0.17 |  | 0.36 | 0.07 |
| MCPP | 1 | 0.23 | 0.38 |  | 0.36 | 0.13 |
| MIBP | 1 | 0.23 | 0.13 |  | 0.36 | 0.26 |
| Equol | 2 | 0.65 | 1.00 |  | 0.26 | 1.00 |
| Cd | 3 | 0.43 | 0.19 |  | 0.76 | 0.07 |
| Pb | 3 | 0.43 | 0.26 |  | 0.76 | 0.14 |
| Hg | 3 | 0.43 | 0.55 |  | 0.76 | 0.79 |

GroupPIP: group posterior inclusion probability; condPIP: conditional posterior inclusion probability. NHANES: National Health and Nutrition Examination Survey. The three groups in BKMR model were phthalate metabolites (group1), equol (group2), and heavy metals (group3). Models were adjusted for age, ethnicity, BMI group, ovary removal, female hormone usage, pregnant status, menopause status, and log-transformed urinary creatinine levels.

**Table S4**. Association between single urinary/blood chemicals and uterine leiomyomata and endometriosis with patients diagnosed more than ten years excluded

| Exposure | OR (95% CI) | | | P for trend |
| --- | --- | --- | --- | --- |
|  | T1 | T2 | T3 |  |
| Uterine leiomyomata | |  |  |  |
| MBP^#^ | 1.00 | 1.28 (0.67, 2.48) | 1.14 (0.55, 2.41) | 0.819 |
| MEP^#^ | 1.00 | 1.28 (0.71, 2.33) | 1.06 (0.56, 2.02) | 0.923 |
| MEHP^#^ | 1.00 | 0.85 (0.47, 1.53) | 0.81 (0.43, 1.52) | 0.520 |
| MBzP^#^ | 1.00 | 1.61 (0.84, 3.13) | 1.37 (0.66, 2.90) | 0.534 |
| MCPP^#^ | 1.00 | 1.45 (0.78, 2.74) | 1.13 (0.55, 2.38) | 0.849 |
| MIBP^#^ | 1.00 | 1.40 (0.75, 2.65) | 1.07 (0.52, 2.24) | 0.991 |
| Equol^#^ | 1.00 | 1.14 (0.62, 2.10) | 1.89 (1.03, 3.50) | 0.034 |
| Cd | 1.00 | 0.95 (0.54, 1.70) | 0.74 (0.41, 1.34) | 0.295 |
| Pb | 1.00 | 1.90 (1.02, 3.66) | 0.88 (0.46, 1.75) | 0.393 |
| Hg | 1.00 | 1.29 (0.70, 2.44) | 1.85 (1.05, 3.35) | 0.033 |
| Endometriosis | |  |  |  |
| MBP^#^ | 1.00 | 1.80 (0.78, 4.34) | 0.80 (0.28, 2.31) | 0.454 |
| MEP^#^ | 1.00 | 0.77 (0.36, 1.63) | 0.66 (0.29, 1.48) | 0.313 |
| MEHP^#^ | 1.00 | 0.75 (0.35, 1.60) | 0.59 (0.25, 1.32) | 0.199 |
| MBzP^#^ | 1.00 | 1.36 (0.57, 3.35) | 1.47 (0.56, 4.02) | 0.468 |
| MCPP^#^ | 1.00 | 0.91 (0.41, 2.07) | 0.56 (0.21, 1.50) | 0.218 |
| MIBP^#^ | 1.00 | 1.35 (0.57, 3.30) | 1.97 (0.77, 5.25) | 0.150 |
| Equol^#^ | 1.00 | 1.47 (0.68, 3.32) | 1.22 (0.53, 2.89) | 0.699 |
| Cd | 1.00 | 2.35 (1.11, 5.32) | 1.36 (0.58, 3.29) | 0.544 |
| Pb | 1.00 | 0.62 (0.27, 1.34) | 1.05 (0.48, 2.32) | 0.960 |
| Hg | 1.00 | 2.15 (1.06, 4.57) | 0.93 (0.39, 2.20) | 0.941 |

T: tertile; OR: odds ratio; CI: confidence interval. Estimated odds ratios (ORs) were calculated while comparing the second, third tertile of each exposure with reference to the first exposure tertile (N = 1152 for uterine leiomyomata and N=1173 for endometriosis). Models were adjusted for age, ethnicity, BMI group, ovary removal, female hormone usage, menopause status, and pregnant status. Models^#^ were further adjusted for log-transformed urinary creatinine levels.

**Table S5**. WQS model to estimate the association between WQS index and uterine leiomyomata and endometriosis with patients diagnosis more than ten years excluded

| Outcomes | OR (95% CI) | P value |
| --- | --- | --- |
| Uterine leiomyomata |  |  |
| b1_positive | 2.52 (1.35, 4.76) | 0.004 |
| b1_negative | 0.75 (0.40, 1.41) | 0.372 |
| Endometriosis |  |  |
| b1_positive | 1.98 (0.80, 4.98) | 0.141 |
| b1_negative | 0.49 (0.20, 1.19) | 0.114 |

OR: odds ratio; CI: confidence interval. OR estimation represents the odds ratios of uterine leiomyomata (N=1152) or endometriosis (N=1173) as one tertile increased in the WQS index. The positive and negative association was estimated respectively. Models were adjusted for age, ethnicity, BMI group, ovary removal, female hormone usage, pregnant status, menopause status, and log-transformed urinary creatinine levels.

**Table S6**. Association between single urinary/blood chemicals and uterine leiomyomata and endometriosis in premenopausal participants (N=1045)

| Exposure | OR (95% CI) | | | P for trend |
| --- | --- | --- | --- | --- |
|  | T1 | T2 | T3 |  |
| Uterine leiomyomata | |  |  |  |
| MBP^#^ | Ref | 1.18 (0.59, 2.39) | 1.13 (0.52, 2.48) | 0.813 |
| MEP^#^ | Ref | 0.97 (0.52, 1.81) | 0.71 (0.36, 1.40) | 0.297 |
| MEHP^#^ | Ref | 0.93 (0.49, 1.77) | 1.10 (0.56, 2.18) | 0.747 |
| MBzP^#^ | Ref | 1.85 (0.94, 3.71) | 1.22 (0.55, 2.75) | 0.809 |
| MCPP^#^ | Ref | 1.52 (0.78, 3.04) | 1.44 (0.67, 3.19) | 0.407 |
| MIBP^#^ | Ref | 1.36 (0.71, 2.65) | 0.91 (0.43, 1.97) | 0.692 |
| Equol^#^ | Ref | 1.22 (0.64, 2.31) | 1.64 (0.88, 3.13) | 0.119 |
| Cd | Ref | 0.97 (0.50, 1.90) | 0.79 (0.44, 1.46) | 0.413 |
| Pb | Ref | 1.31 (0.66, 2.72) | 1.05 (0.52, 2.24) | 0.937 |
| Hg | Ref | 1.03 (0.52, 2.04) | 1.77 (0.97, 3.33) | 0.045 |
| Endometriosis | |  |  |  |
| MBP^#^ | Ref | 1.62 (0.71, 3.80) | 1.23 (0.47, 3.31) | 0.823 |
| MEP^#^ | Ref | 0.65 (0.30, 1.34) | 0.74 (0.34, 1.56) | 0.435 |
| MEHP^#^ | Ref | 0.53 (0.24, 1.09) | 0.61 (0.28, 1.29) | 0.202 |
| MBzP^#^ | Ref | 2.16 (0.93, 5.26) | 2.68 (1.05, 7.23) | 0.055 |
| MCPP^#^ | Ref | 1.22 (0.56, 2.73) | 0.91 (0.36, 2.33) | 0.770 |
| MIBP^#^ | Ref | 1.13 (0.50, 2.56) | 1.78 (0.76, 4.29) | 0.169 |
| Equol^#^ | Ref | 1.17 (0.57, 2.40) | 0.79 (0.36, 1.73) | 0.527 |
| Cd | Ref | 1.09 (0.49, 2.38) | 1.12 (0.57, 2.28) | 0.748 |
| Pb | Ref | 1.04 (0.51, 2.15) | 0.91 (0.41, 2.07) | 0.819 |
| Hg | Ref | 1.65 (0.83, 3.38) | 1.06 (0.49, 2.31) | 0.886 |

T: tertile; OR: odds ratio; CI: confidence interval. Estimated odds ratios (ORs) were calculated while comparing the second, third tertile of each exposure with reference to the first exposure tertile. Models were adjusted for age, ethnicity, BMI group, ovary removal, female hormone usage, and pregnant status. Models^#^ were further adjusted for log-transformed urinary creatinine levels.


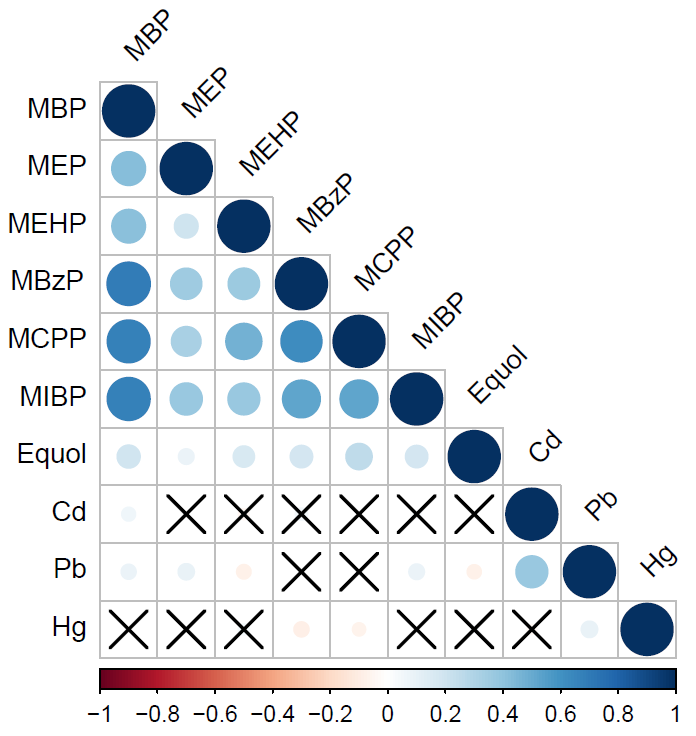


**Figure S1**. *Pearson*’s correlations among ten chemical exposures or metabolites (N = 1204), NHANES, 2001–2006. Correlations without statistical significance (P ≥ 0.05) were marked with crosses.


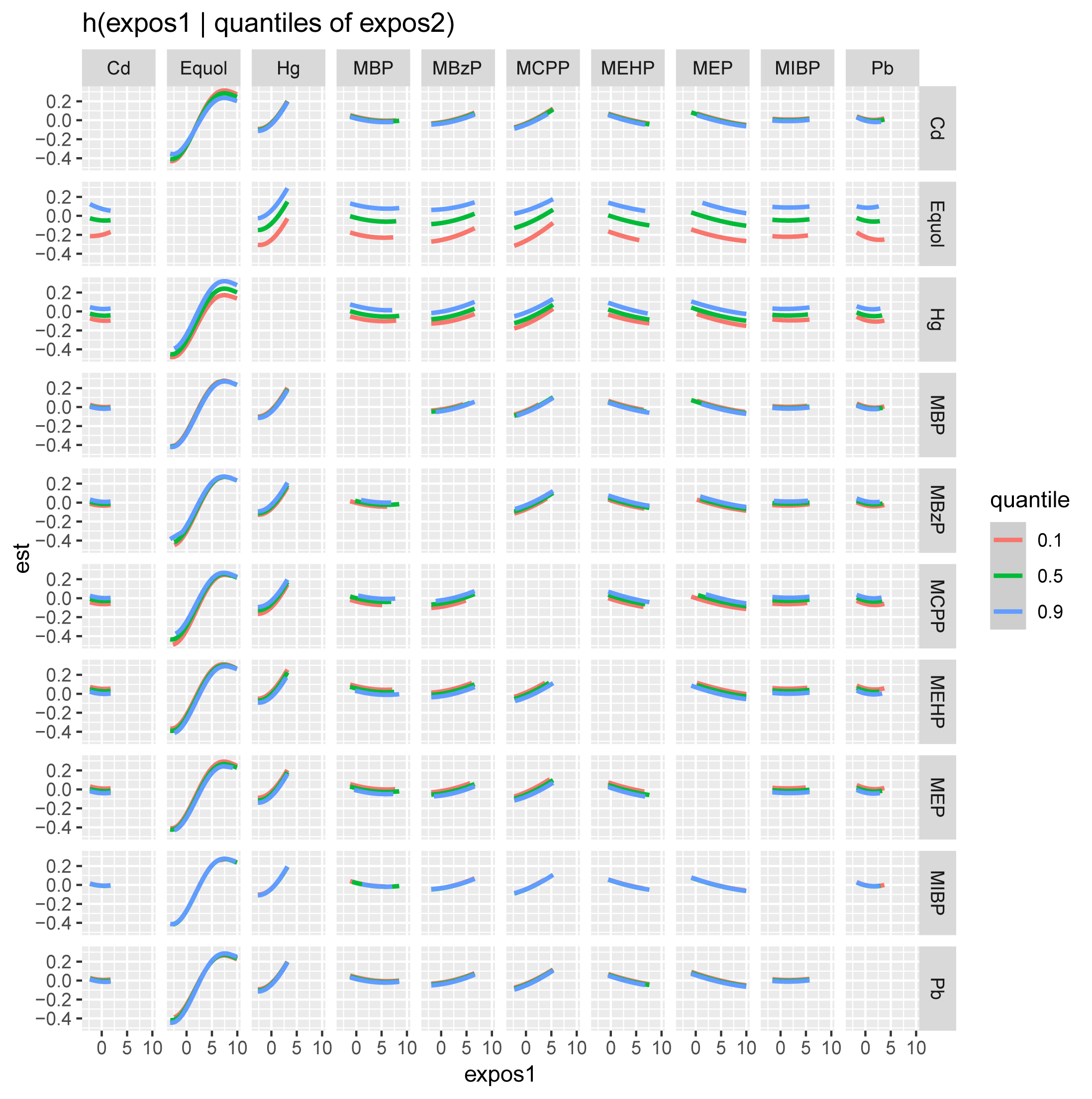


**Figure S2**. Association between exposure 1 with uterine leiomyomata, while fixing exposure 2 at different percentiles (here are the 10th, 50th, and 90th percentiles) all the others at their median levels. Model was adjusted for age, ethnicity, BMI group, ovary removal, female hormone usage, pregnant status, menopause status, and log-transformed urinary creatinine levels.


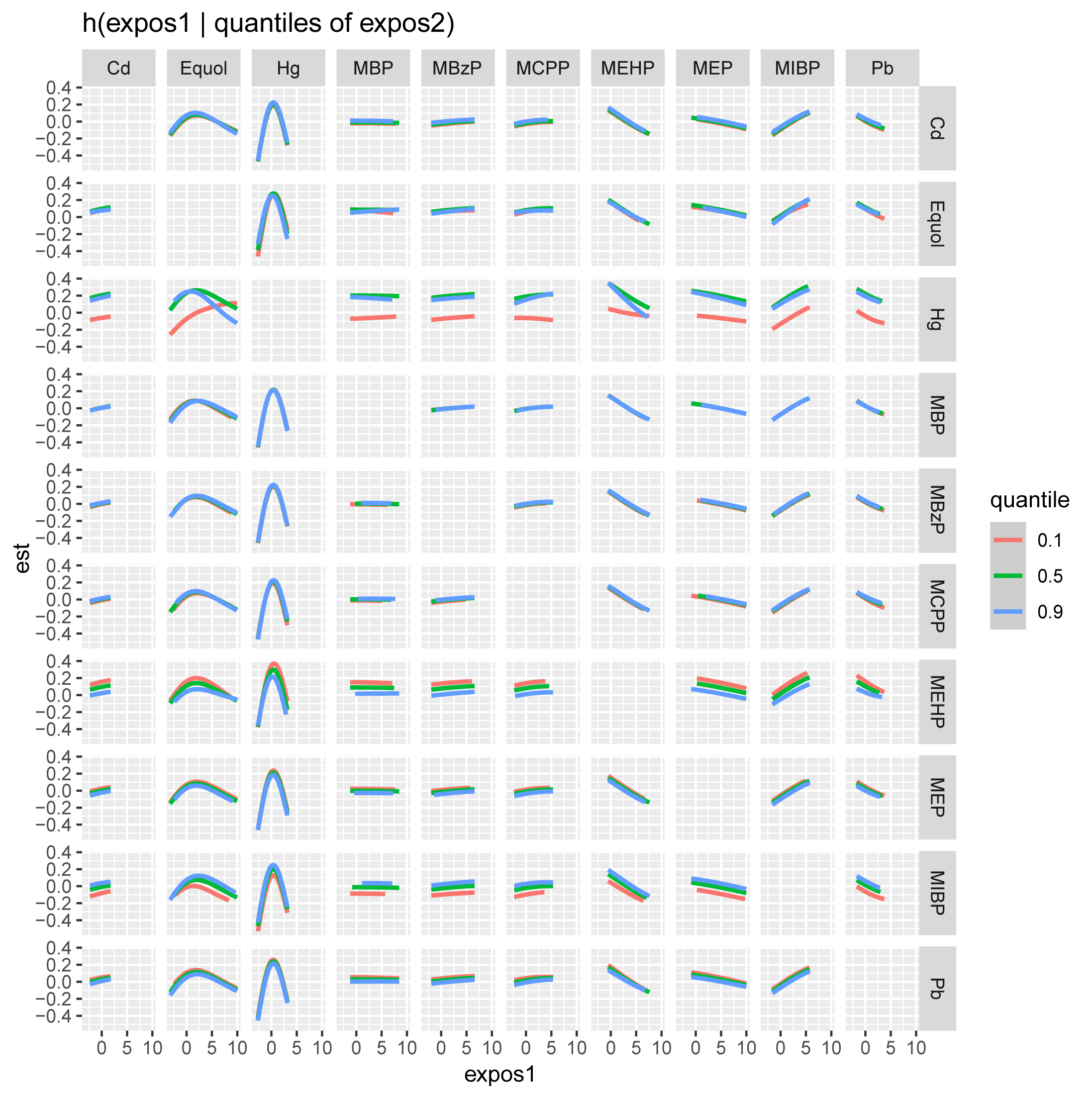


**Figure S3**. Association between exposure 1 with endometriosis, while fixing exposure 2 at different percentiles (here are the 10th, 50th, and 90th percentiles) all the others at their median levels. Model was adjusted for age, ethnicity, BMI group, ovary removal, female hormone usage, pregnant status, menopause status, and log-transformed urinary creatinine levels.


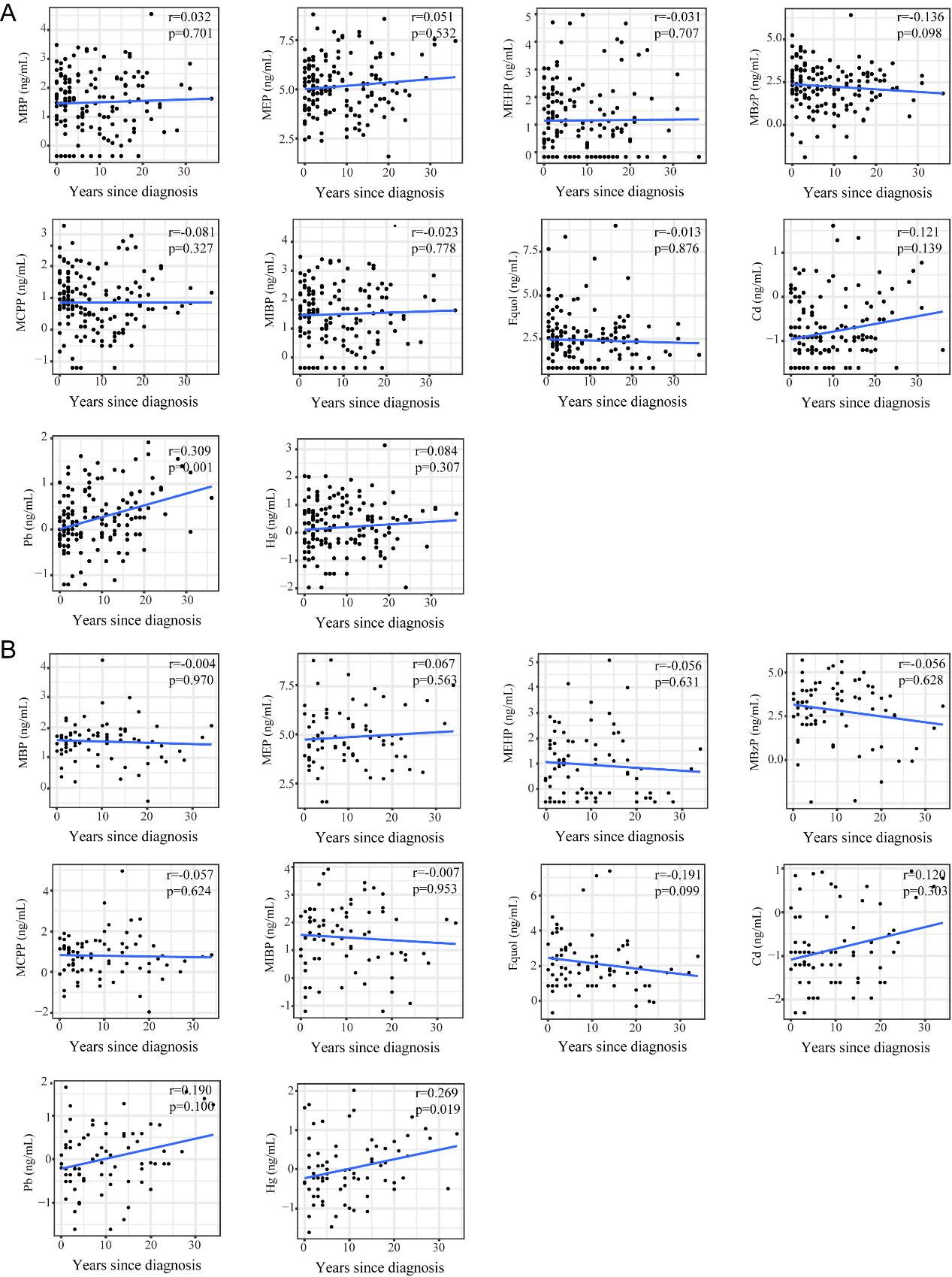


**Figure S4.** Spearman correlation between chemical exposure levels and the number of years after uterine leiomyomata (A) and endometriosis (B) diagnosis. The concentration of chemical exposure level was ln-transformed.


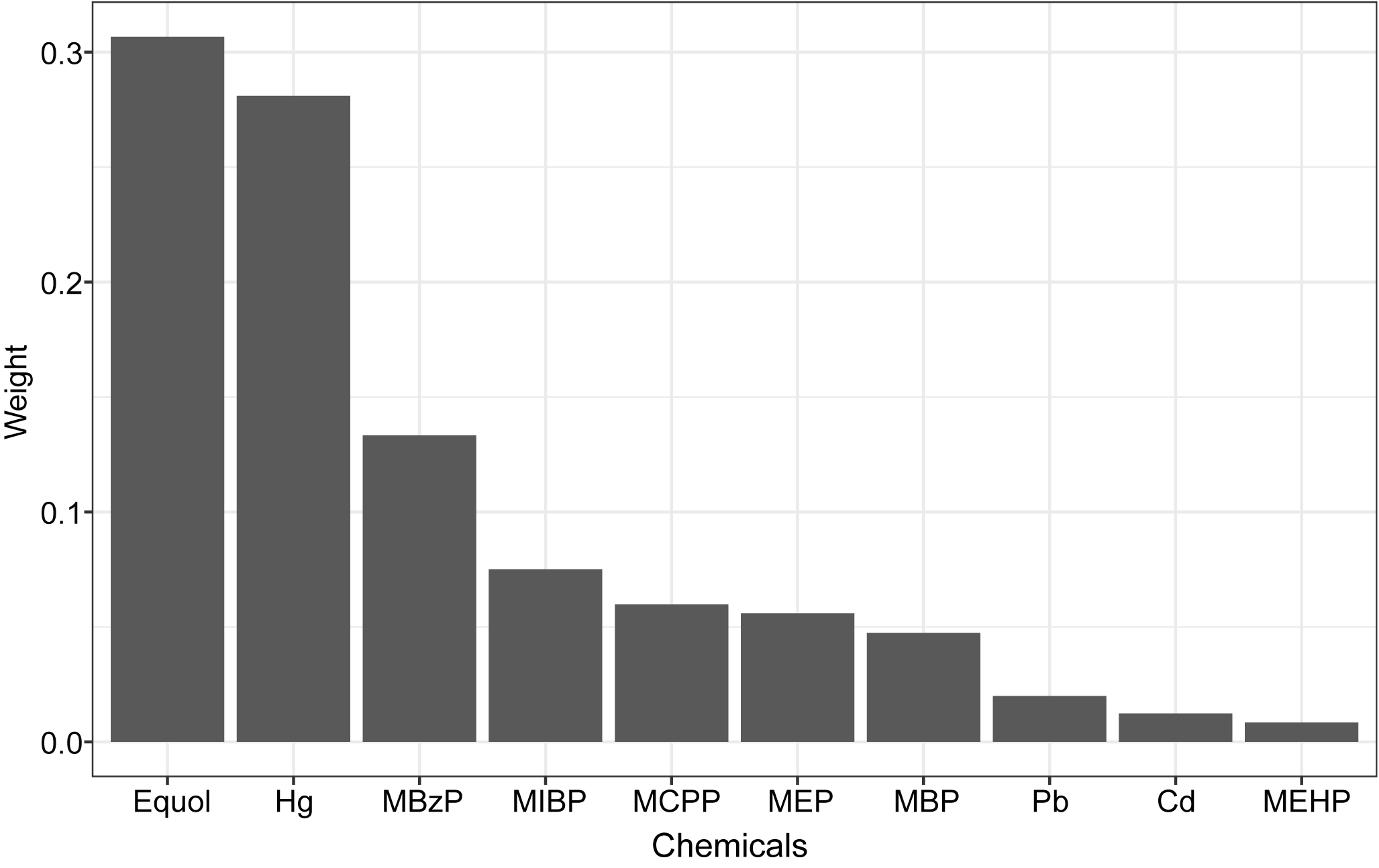


**Figure S5.** WQS model regression index weights for the uterine leiomyomata, in which the women diagnosed with uterine leiomyomata more than 10 years were excluded. Model was adjusted for age, ethnicity, BMI group, ovary removal, female hormone usage, pregnant status, and log-transformed urinary creatinine levels.


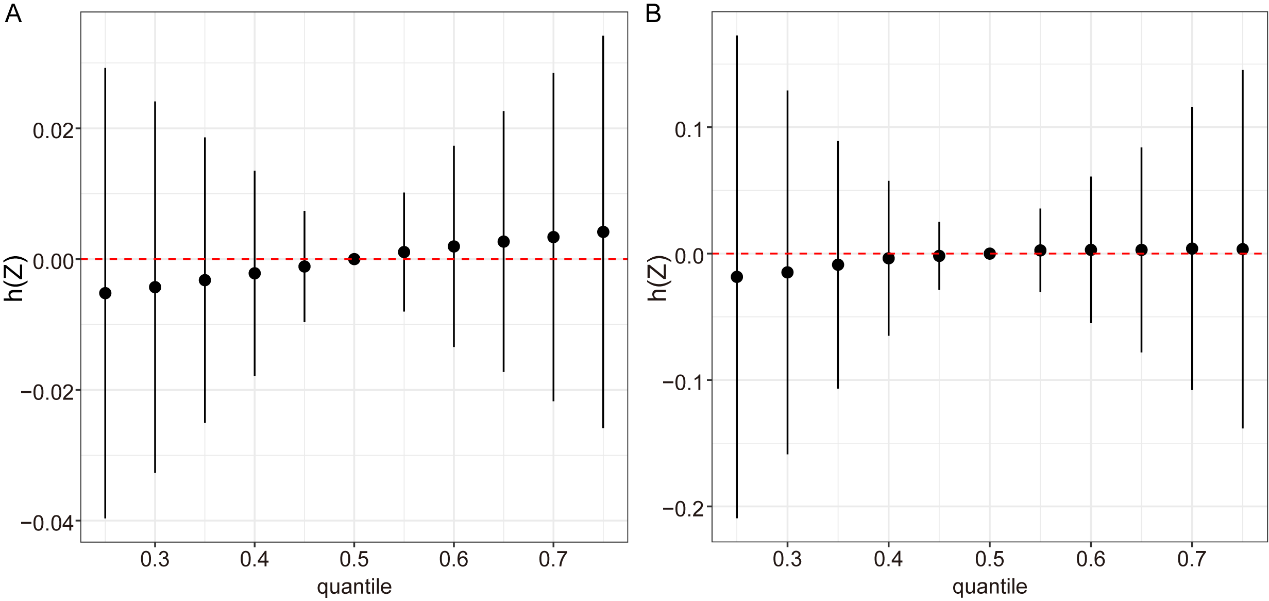


**Figure S6**. Overall risk (95% CI) of the mixture on uterine leiomyomata (A) and endometriosis (B), in which the women diagnosed with uterine leiomyomata more than 10 years were excluded when comparing all the chemicals at different percentiles with their median level. Models were adjusted for age, ethnicity, BMI group, ovary removal, female hormone usage, pregnant status, and log-transformed urinary creatinine levels.


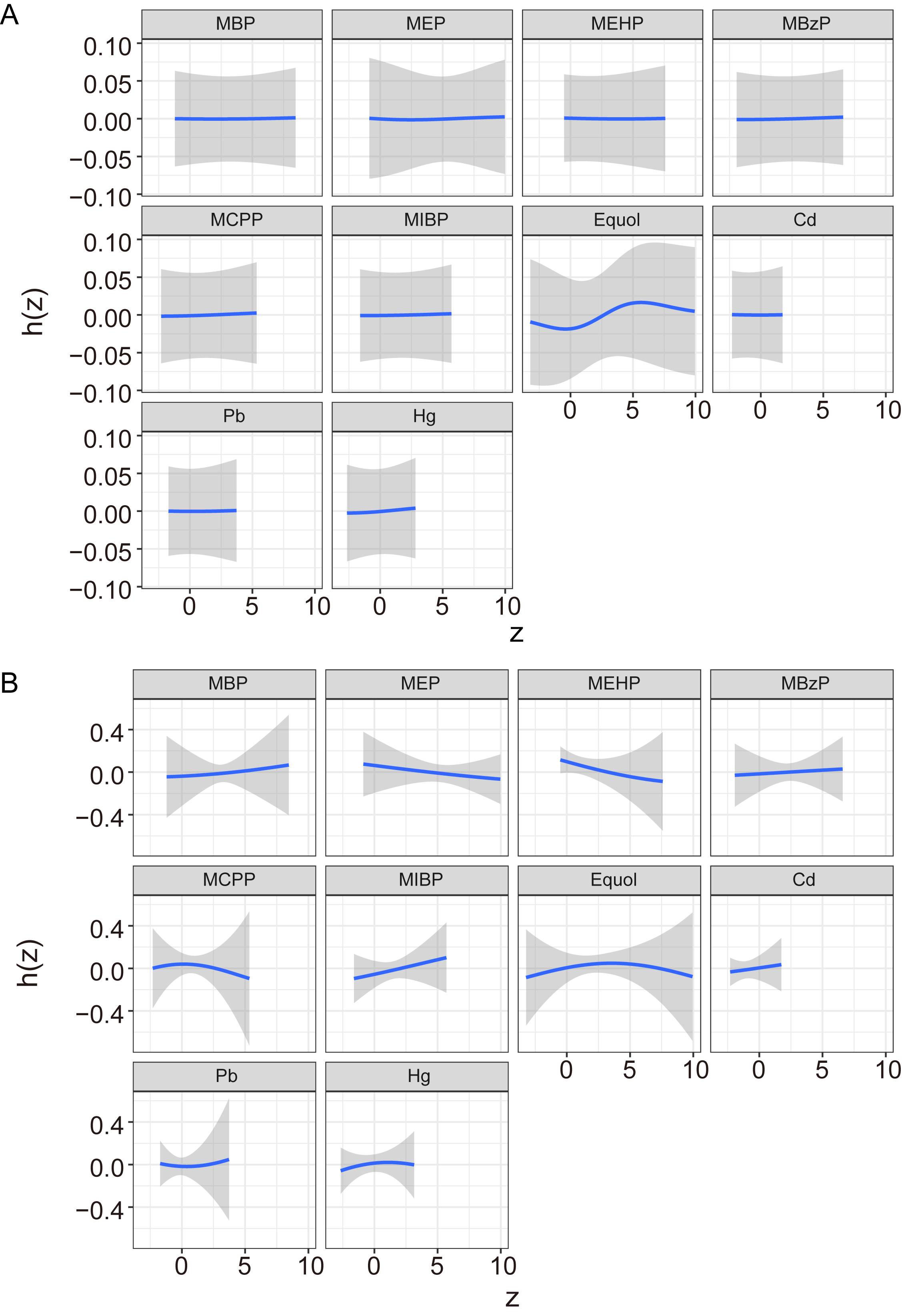


**Figure S7**. Univariate exposure-response function (95% CI) between selected chemical concentrations and uterine leiomyomata (A) and endometriosis (B), in which the women diagnosed with uterine leiomyomata more than 10 years were excluded while fixing other chemicals at their median level. Models were adjusted for age, ethnicity, BMI group, ovary removal, female hormone usage, pregnant status, menopause status, and log-transformed urinary creatinine levels.


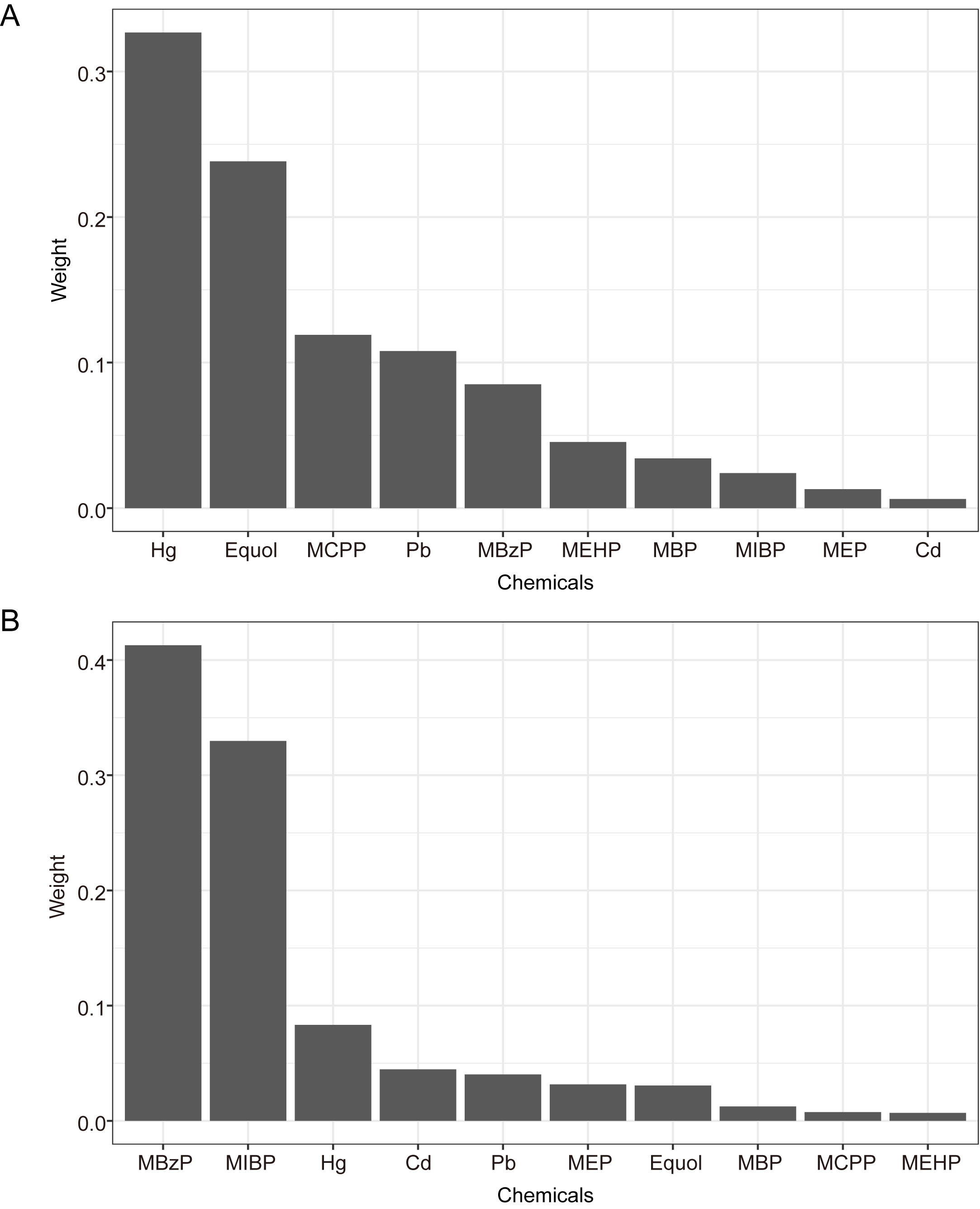


**Figure S8.** The weights of each chemical in positive WQS model regression index for uterine leiomyomata (A) and endometriosis (B) in premenopausal participants (N=1045). Models were adjusted for age, ethnicity, BMI group, ovary removal, female hormone usage, pregnant status, and log-transformed urinary creatinine levels.


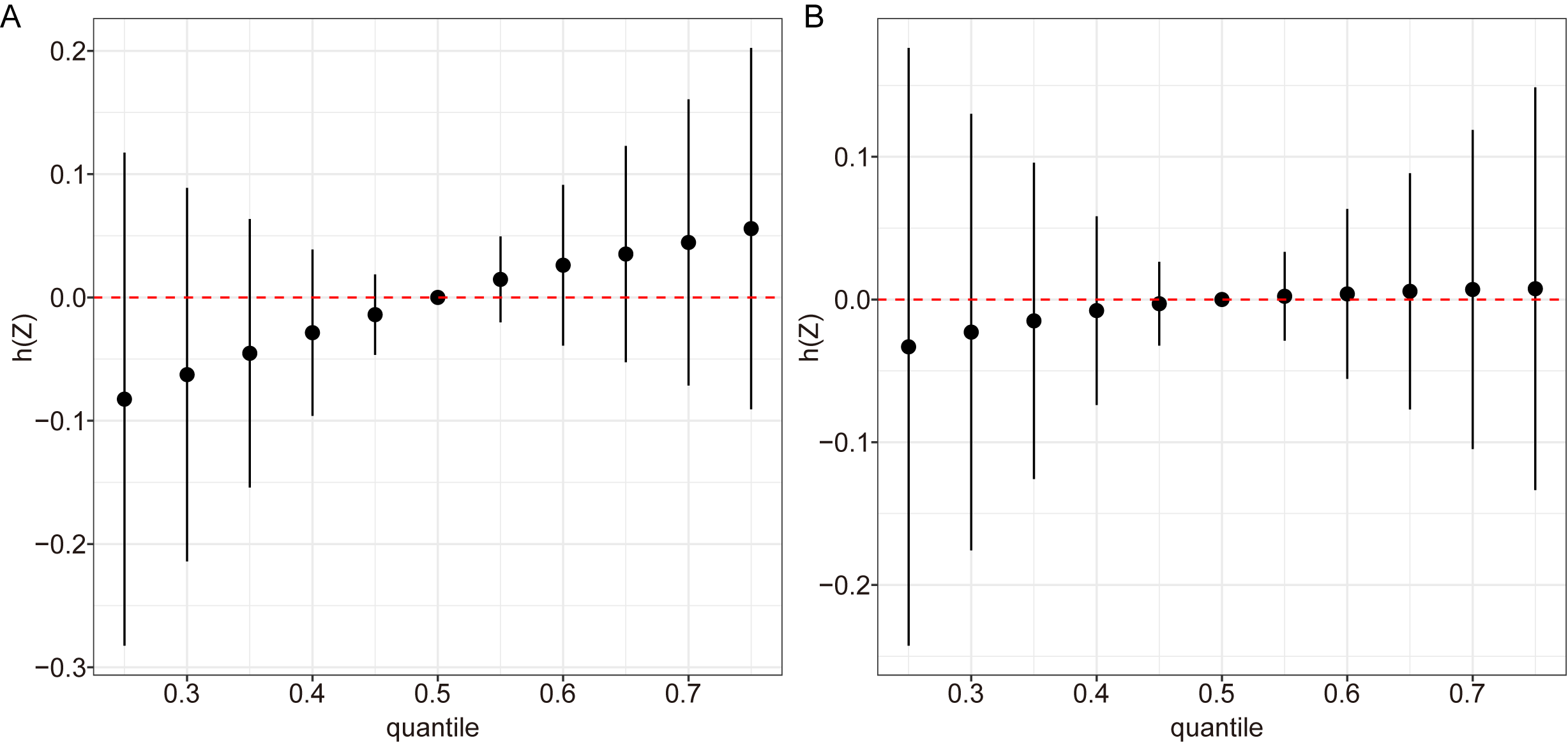


**Figure S9**. Overall risk (95% CI) of the mixture on uterine leiomyomata (A) and endometriosis (B) in premenopausal participants, when comparing all the chemicals at different percentiles with their median level. Models were adjusted for age, ethnicity, BMI group, ovary removal, female hormone usage, pregnant status, and log-transformed urinary creatinine levels.


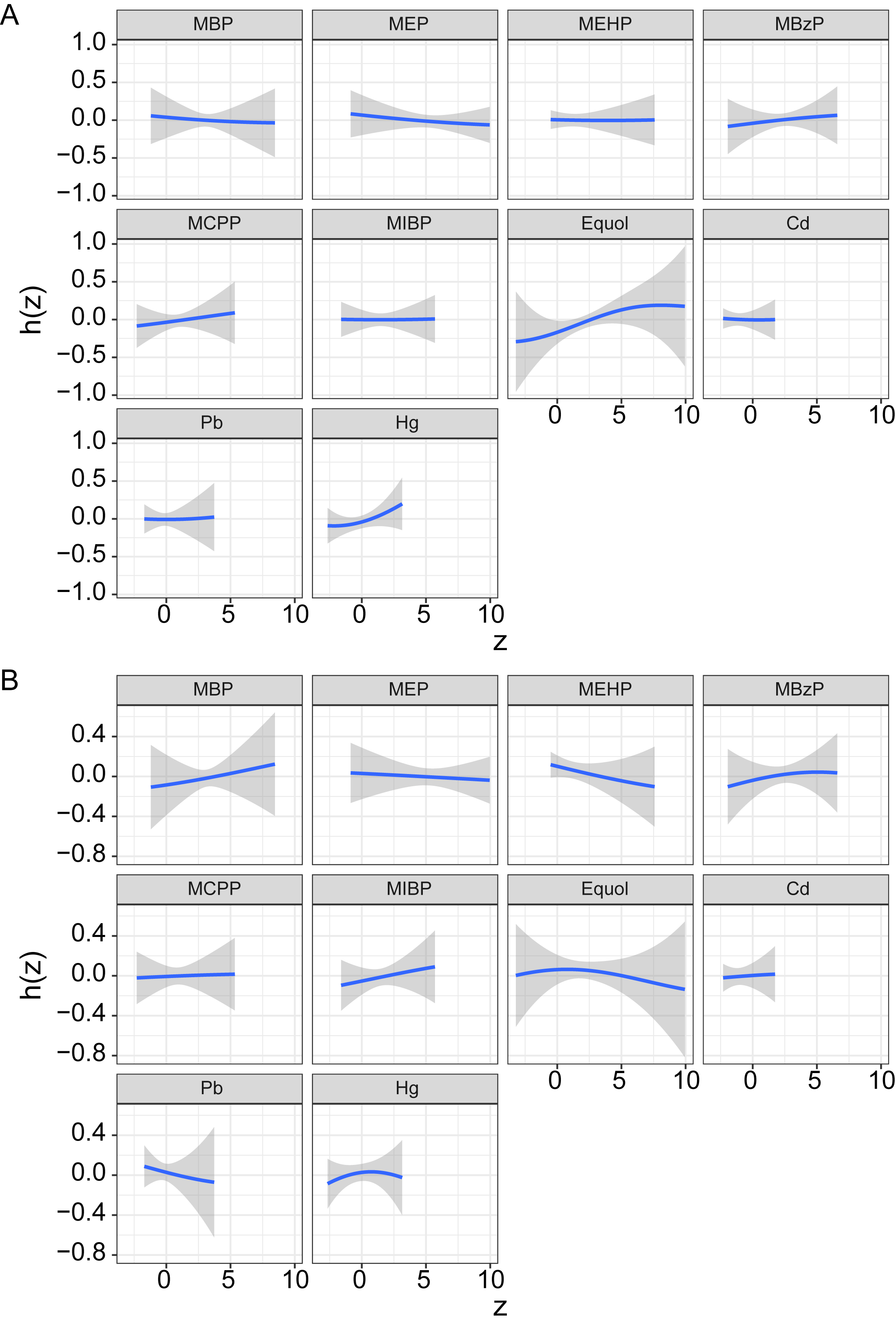


**Figure S10**. Univariate exposure-response function (95% CI) between selected chemical concentrations and uterine leiomyomata (A) and endometriosis (B) in premenopausal participants, while fixing other chemicals at their median level. Models were adjusted for age, ethnicity, BMI group, ovary removal, female hormone usage, pregnant status, and log-transformed urinary creatinine levels.
